# Self-rated Health Status of the Adult Population in Addis Health and Demographic Surveillance System (Addis-HDSS), Addis Ababa, Ethiopia

**DOI:** 10.4314/ejhs.v34i2.6S

**Published:** 2024-12

**Authors:** Dagmawit Tewahido, Semira Abdelmenan, Firehiwot Workneh, Workagegnhu Tarekegn, Kalkidan Yibeltal, Hana Sime, Hanna Y Berhane, Sitota Tsegaye, Nebiyou Fasil, Dongqing Wang, Uttara Partap, Wafaie Fawzi, Meaza Demissie, Alemayehu Worku, Yemane Berhane

**Affiliations:** 1 Department of Nutrition and Behavioral Sciences, Addis Continental Institute of Public Health; 2 Department of Epidemiology and Biostatistics, Addis Continental Institute of Public Health; 3 Department of Reproductive Health and Population, Addis Continental Institute of Public Health; 4 Department of Global Health and Health Policy, Addis Continental Institute of Public Health; 5 Department of Global and Community Health, College of Public Health, George Mason University, Fairfax, Virginia, United States of America; 6 Department of Global Health and Population, Harvard T.H. Chan School of Public Health, Harvard University, Boston, Massachusetts, United States of America

**Keywords:** Self-rated health, adults, Addis Ababa, HDSS

## Abstract

**Background:**

Self-rated health (SRH) status is a subjective assessment of one's health condition and can serve as a reliable indicator of a community's overall health. This study aimed to evaluate the SRH status of communities and its association with socio-demographic and health-related variables at the population level.

**Methods:**

Data were obtained from the Addis Health and Demographic Surveillance System. SRH was assessed through a single question: “In general, would you say that your health is excellent, very good, good, fair, or poor?” These five categories were transformed into two groups: “Good SRH” and “Poor SRH.” Bivariate and multivariate logistic regression analyses examined associations between SRH status and socio-demographic and health-related characteristics.

**Results:**

A total of 46,483 adults (aged 18 and above) were included in the study. Of these, 4,377 (9.42%) participants reported poor SRH status. Male sex (OR 0.87; 95% CI: 0.80 – 0.94), higher educational level (OR 1.90; 95% CI: 1.67 – 2.17), and the highest wealth index (OR 1.76; 95% CI: 1.55 – 2.00) were significantly associated with good SRH status, while older age (OR 0.15; 95% CI: 0.12 – 0.18) and the presence of any chronic illness (OR 0.08; 95% CI: 0.07 – 0.09) were directly and significantly associated with poor SRH status.

**Conclusion:**

Poor SRH status was more prevalent among females, older individuals, those with lower education, lower wealth index, and those with chronic illnesses. These findings highlight the need for robust health facilities and support systems for women and the elderly population.

## Introduction

Self-rated health (SRH) is an individual's subjective evaluation of their own health status, which is assumed to capture multiple dimensions of one's health (1, 2). SRH status is considered a valuable indicator for understanding the type and distribution of health issues within a population (3, 4). The SRH questionnaire is a simple and easily understandable tool that can be integrated into large population-based surveys with minimal additional time required (5-7). As a measure of subjective health, SRH has gained substantial recognition in health studies and is used to predict future health outcomes. Individuals with poor SRH are at an increased risk of developing chronic health problems, disability, or premature death (8). Additionally, it has been suggested that SRH can reflect an individual's social and economic circumstances, and can be used to address health disparities across different socioeconomic groups (9, 10).

Self-rated health is typically assessed with a single question on a Likert scale ranging from 1 to 5, where 1 indicates the worst and 5 indicates excellent health status (4). However, SRH is a complex concept that takes into account various factors, including an individual's physical, psychological, interpersonal, and functional aspects, as well as cultural and personal opinions and health behaviors (11).

A key limitation of SRH is that it is more prone to contextual effects than objectively evaluated health status (12, 13). SRH is also highly sensitive to sociocultural differences across countries, making it less useful for cross-country comparisons (14). Variations in survey question formulation and translation, especially in terms of scale, can also impact the comparability of responses (15). Despite these limitations, SRH remains a robust measure of morbidity, helping to identify socio-demographic, environmental, and clinical factors that inform health systems and policies (16, 17).

The way self-rated health is understood and perceived within a specific population is crucial for proper interpretation, as it is commonly understood across the global community (18). SRH is often associated with socio-demographic characteristics such as age and sex (19-21). It also reflects regional patterns based on the education and economic status of populations (22).

SRH in Sub-Saharan Africa remains largely unexplored (23, 24). Understanding the health status of urban populations in Africa has become a priority due to rapid urbanization and demographic changes (25, 26). This study aims to assess the SRH status of adults and identify associated socio-demographic and health-related factors in Addis Ababa, the capital city of Ethiopia.

## Methods

**Figure 1:**
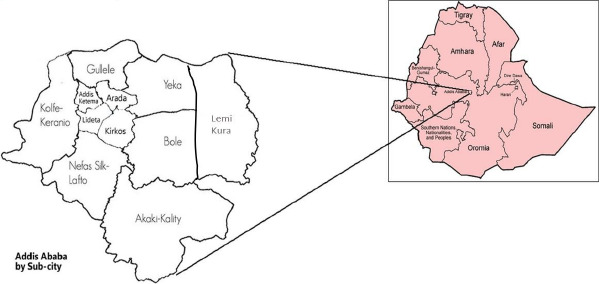
Addis Ababa by sub-cities: Location of Yeka sub-city

**Study setting and design**: This study utilized census data from the Addis Health and Demographic Surveillance System (Addis-HDSS) in Addis Ababa, Ethiopia. According to UN estimates, Addis Ababa has a population of 5.2 million, with a land area of 527 square kilometers and a population density of 5,165 individuals per square kilometer. The population is growing at an annual rate of 4.4%, making Addis Ababa one of the fastest-growing cities in the world (27). The Addis-HDSS site, established by the Addis Continental Institute of Public Health (ACIPH) in December 2022, is located in the Yeka sub-city. The site covers 6 Woredas (districts) and 240 enumeration areas (Figure 1).

**Study population**: The study included 46,483 adults who participated in the Addis-HDSS census during December 2022 and January 2023. All permanent residents who responded to the SRH question were included. The study included data from 46,483 adults living in 30,533 households. Household heads and spouses were the primary respondents, with individual household heads interviewed when only one was present.

**Data collection and procedures**: Data were collected using a structured, interviewer-administered, tablet-based questionnaire that addressed SRH and associated factors, including socio-demographic, economic, and health-related variables. Trained enumerators conducted house-to-house interviews. The study tools were pretested in Woreda 10, a similar area in the Yeka sub-city. The interviews were conducted in Amharic, the local language.

**Measurement**: SRH was assessed using a single question: “In general, would you say that your health is excellent, very good, good, fair, or poor?” These five categories were collapsed into two groups: “Good SRH” (for “excellent” and “very good”) and “Poor SRH” (for “good,” “fair,” and “poor”) (17, 28).

The socio-demographic variables considered for this study included age (18-29, 30-39, 40-49, 50-59, 60-69, 70+), gender (male, female), educational level (no formal education, primary education, secondary education, higher education), marital status (single, divorced, married, separated, widowed), and wealth index (lowest, second, middle, fourth, highest). The wealth index was constructed using Principal Component Analysis (PCA), incorporating factors like house type, ownership, number of bedrooms, kitchen, water source, toilet availability, toilet-sharing status, shower facility, car ownership, and regular bank savings.

Health-related variables included physical disability, mental disability, and chronic illness. Physical and mental disability were assessed with yes/no questions. Chronic illness was identified by asking participants if they had any of the following conditions: hypertension, diabetes mellitus, asthma, cancer, or other chronic diseases.

**Analysis**: Data were analyzed using STATA 14.0. Descriptive statistics were used to calculate frequencies and proportions for categorical variables. Pearson's chi-square test assessed the association between SRH and socio-demographic and health-related factors. Binary and multivariate logistic regression models were used to examine associations between SRH and socio-demographic variables such as sex, age, education, wealth index, and chronic illness. Participants with physical and mental disabilities were excluded from the logistic regression analysis due to the small number of cases. The full model included chronic illness as a variable. A sensitivity analysis was also performed by excluding individuals with chronic illnesses, reducing the sample size to 38,887. Multicollinearity among the factors was assessed using variance inflation factors (VIF), which showed a mean VIF of 1.81, indicating slight but acceptable multicollinearity. Adjusted odds ratios (AOR) with 95% confidence intervals (CI) were used to describe the strength of associations. Statistical significance was set at p < 0.05.

**Ethical considerations**: Ethical clearance was obtained from the ACIPH Ethical Review Committee (ACIPH/IRB/003/2022) and the Addis Ababa Health Bureau Ethical Review Committee. Participants (household heads) were fully informed about the study's purpose, and written informed consent was obtained before data collection.

## Results

The study included 46,483 adults (≥18 years), with 57% females. The mean age was 42.9 years (±15.4). About one-third of participants (14,676, 31.6%) had higher education, while 5,512 (11.9%) had no formal education. The majority were married (33,140, 71.29%) (Table 1). 7,596 (16.3%) participants reported chronic illness, while 1,389 (3.0%) had a physical disability and 465 (1.0%) had a mental disability (Table 2).

**Table 1:** Socio-demographic characteristics (N=46,483)

Socio-demographic characteristics		Number (%)
Sex	Male	20,010 (43.0%)
	Female	26,473 (57.0%)
Age in years	Mean age (mean ± standard deviation)	42.9 (±15.4)
	18-29	9,086 (19.5%)
	30-39	14,599 (31.4%)
	40-49	9,381 (20.2%)
	50-59	5,599 (12.0%)
	60-69	4,156 (8.9%)
	70+	3,662 (7.9%)
Educational status	No formal education	5,512 (11.9%)
	Primary education	12,473 (26.8%)
	Secondary education	13,822 (29.7%)
	Higher education	14,676 (31.6%)
	Single	4933 (10.61%)
Marital status	Married	33,140 (71.29%)
	Divorced	2,087 (4.49%)
	Widowed	5,183 (11.15%)
	Separated	1,140 (2.45%)
wealth index in quintiles	lowest	9,762 (21.0%)
	second	8,417 (18.1%)
	middle	9,314 (20.0%)
	fourth	9,359 (20.1%)
	highest	9,631 (20.7%)

**Table 2:** Health-related variables (N=46,483)

Variable	Response	Number(%)
Any chronic illness	No	38,887 (83.7%)
	Yes	7,596 (16.3%)
physical disability	No	45,094 (97.0%)
	Yes	1,389 (3.0%)
mental disability	No	46,018 (99.0%)
	Yes	465 (1.0%)

**Figure 2:**
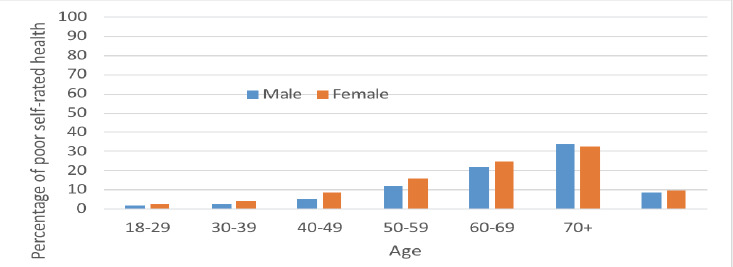
Self-rated Health Status by age (in years) and sex

Overall, 4,377 (9.42%) participants reported poor SRH. Good SRH was reported by 91.42% of males and 89.95% of females. As age increased by a decade, the proportion reporting good SRH decreased. About 66.2% of those aged 70 and above reported good SRH, compared to about 98% in the 18-29 age group (Figure 2).

The multivariable logistic regression analysis (Model 1) revealed that males had slightly higher odds of good SRH than females (OR: 1.07, 95% CI: 0.99–1.16). Those aged 70+ had much lower odds of good SRH (OR: 0.15, 95% CI: 0.12–0.18) compared to the 18-29 age group. Participants with higher education had nearly 2 times higher odds of good SRH compared to those with no formal education (OR: 1.90, 95% CI: 1.67–2.17). The wealthiest individuals had 1.7 times higher odds of good SRH compared to the poorest (OR: 1.76, 95% CI: 1.55–2.00). Those with chronic illnesses had significantly lower odds of good SRH (OR: 0.08, 95% CI: 0.07–0.09). In contrast, the sensitivity analysis (Model 2) revealed increased odds of good SRH for males and consistent negative associations with increasing age, especially in older age groups. Education continued to show a strong positive association with SRH, while the highest wealth quintile was strongly associated with good SRH (Table 3).

**Table 3:** Self-rated Health status and associated factors

Background factors		Crude OR (95% CI)	Model 1 (N=46,483) AOR	Model 2 (N=38,887) AOR
Sex	Female	1.0	1.0	1.0
	Male	1.19*[1.11-1.26]	1.07 [0.99-1.16]	1.15*[1.02-1.30]
Age	18-29	1.0	1.0	1.0
(years)	30-39	0.62*[0.53-0.73]	0.69* [0.58-0.82]	0.70*[0.56-0.86]
	40-49	0.32*[0.27-0.37]	0.47* [0.39-0.55]	0.43*[0.35-0.54]
	50-59	0.14*[0.12-0.16]	0.30* [0.25-0.36]	0.24*[0.19-0.30]
	60-69	0.07*[0.06-0.08]	0.22* [0.18-0.26]	1.17*[0.13-0.22]
	70+	0.04* [-.04-0.55]	0.15* [0.12-0.18]	0.10*[0.08-0.13]
Education	No formal education	1.0	1.0	1.0
	Primary education	2.12*[1.94-2.30]	1.20*[1.08-1.34]	1.48*[1.26-1.73]
	Secondary education	3.34*[3.05-3.66]	1.13*[1.17-1.49]	1.64*[1.38-1.96]
	Higher education	5.08*[4.61-5.61]	1.90*[1.67-2.17]	2.48*[2.01-3.05]
Wealth Index	Lowest	1.0	1.0	
	Second	0,92[0.83-1.01]	1.13*[1.0-1.27]	1.03[0.87-1.22]
	Middle	0.97[0.88-1.07]	1.29*[1.14-1.14]	1.17[0,98-1.38]
	Fourth	0.89*[0.81-0.98]	1.58*[1.40-1.78]	1.50* [1.25-1.80]
	Highest	0.88*[9.60-11.04]	1.76*[1.55-2.0]	2.14*[1.74-2.64]
Any chronic illness	No	1.0	1.0	-
	Yes	0.05*[0.04-0.05]	0.08*[0.07-0.90]	-

* p < 0.05

Model 1: The full model including the chronic illness variable to capture its effect on SRH among a sample of 46,483 individuals

Model 1: Sensitivity analysis to see the impact of chronic illnesses by re-running logistic regression excluding individuals with chronic illnesses

## Discussion

The study, based on 46,483 participants, found that 9.42% reported poor SRH. Key factors associated with poor SRH included female sex, older age, lower education and wealth, and chronic illness. These findings of female sex and older age being associated with poor SRH are consistent with other studies in different settings (29, 30). Poor health among older women is particularly concerning, as they often have less mobility and are more likely to experience functional and cognitive impairments. In Ethiopia, women's caregiving roles and reproductive responsibilities may negatively impact their health, especially as they age (31-33). Men's higher income, education, and economic status are associated with better SRH (34). The strong association between wealth and health is likely due to better access to health services, healthier living conditions, and more resources to manage chronic conditions (35). It's important to consider these socio-economic factors when designing health interventions to address health disparities in urban populations.

This study provides important insights into the SRH status of urban adults in Addis Ababa. It highlights the need to consider socio-demographic factors, especially sex, age, education, and wealth, when addressing urban health disparities. Health programs targeting chronic illness and providing access to quality health care for disadvantaged groups are necessary to improve health outcomes in this rapidly growing urban population.
